# P-698. Evaluating Modes of Influenza Transmission (EMIT-2): An Ongoing Controlled Human Influenza Virus Infection Transmission Trial (CHIVITT)

**DOI:** 10.1093/ofid/ofae631.894

**Published:** 2025-01-29

**Authors:** Jianyu Lai, Kristen K Coleman, Sheldon Tai, Filbert Hong, Isabel Sierra Maldonado, Yi Esparza, Hamed Sobhani, Jelena Srebric, Proma Bhattacharya, Don L DeVoe, Wilbur Chen, Donald K Milton

**Affiliations:** University of Maryland, College Park, College Park, Maryland; University of Maryland School of Public Health, College Park, Maryland; University of Maryland, College Park, Maryland; University Of Maryland School of Public Health, College Park, Maryland; University of Maryland, College Park, Maryland; University of Maryland College Park, School of Public Health, College Park, Maryland; Department of Mechanical Engineering, University of Maryland, College Park, Maryland; University of Maryland College Park, College Park, Maryland; University of Maryland College Park, College Park, Maryland; University of Maryland College Park, College Park, Maryland; University of Maryland School of Medicine, Baltimore, Maryland; University of Maryland, College Park, Maryland

## Abstract

**Background:**

The relative importance of inhalation, spray, and touch transmission remains poorly understood. We implemented a randomized controlled trial incorporating community-acquired cases using behavioral, personal protective equipment, and environmental interventions as tools to understand the pathway of influenza transmission.

**Figure 1.**
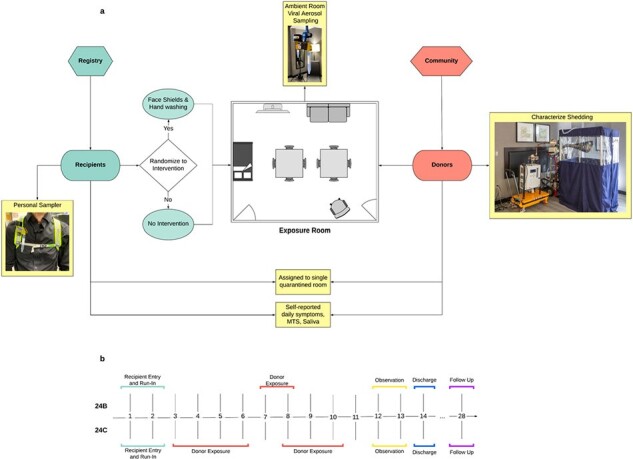
Study design and timeline of cohorts with naturally infected influenza Donors

**Methods:**

We recruited healthy volunteer Recipients and influenza Donors with PCR-confirmed community-acquired infection to a hotel quarantine. We randomized healthy volunteers to Intervention (hand hygiene and face shield) and Control Recipients. Donors and Recipients interacted in an “Event Room” with controlled ventilation (0.2–0.5 air changes per hour) and relative humidity (20-40%). We collected ambient air and personal bioaerosol exposure samples using NIOSH BC-251 samplers. We also deployed a novel cascade to liquid media bioaerosol sampler. Donors provided exhaled breath samples using a Gesundheit-II (G-II) sampler. We analyzed samples using dPCR and florescent focus assay.

**Figure 2.**
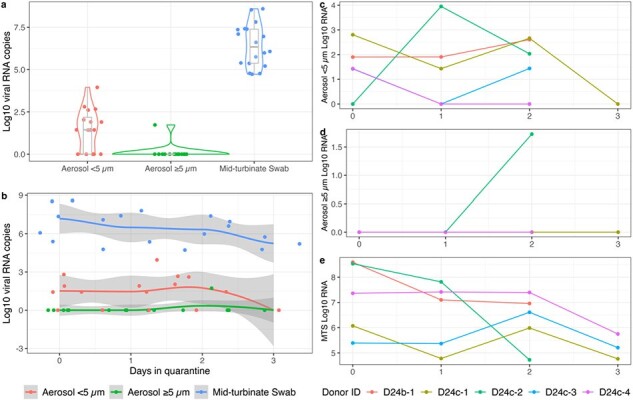
Viral RNA load in GII samples and MTS

**Results:**

We ran four cohorts (February 2023 and January-February 2024); two with naturally infected influenza Donors (Figure 1). We exposed 11 Recipients (mean age: 36; 55% female) to 5 influenza Donors (mean age: 21; 80% female). Preliminary results show that eleven G-II fine (< 5µm), one G-II coarse (≥5µm) (Figure 2), four NIOSH ambient air (three ≥4µm and one 1-4µm), and three NIOSH personal bioaerosol exposure samples (two 1-4µm and one less than 1µm) were PCR positive. Virus was cultured from an ambient bioaerosol sample. No Recipient developed influenza-like illness or PCR-positive swabs; serology is pending.

**Conclusion:**

We demonstrated that it is feasible to recruit Donors with new onset community-acquired influenza infections and expose Recipients under highly controlled conditions. Although initial experiments did not produce PCR-positive secondary infections, much will be learned by analyzing the immunity of the exposed Recipients and the inhaled dose of virus. This unique randomized controlled trial will provide a definitive assessment of the role of inhalation transmission and critical data on which to build effective interventions to prevent transmission.

**Disclosures:**

**Donald K. Milton, MD, DrPH**, Lumen Bioscience, Inc: Advisor/Consultant|Lumen Bioscience, Inc: Stocks/Bonds (Private Company)

